# Immunological Role of the Maternal Uterine Microbiota in Postpartum Hemorrhage

**DOI:** 10.3389/fimmu.2020.00504

**Published:** 2020-03-31

**Authors:** Maria F. Escobar, Maria A. Hincapie, Juan S. Barona

**Affiliations:** ^1^Fundación Valle del Lili, Gynecologist and Obstetrician, High Complexity Obstetrics Unit, Cali, Colombia; ^2^Department of Gynecology and Obstetrics, Universidad ICESI, Cali, Colombia; ^3^Faculty of Health Sciences, Universidad ICESI, Cali, Colombia; ^4^Department of Gynecology and Obstetrics, Universidad ICESI, Cali, Colombia

**Keywords:** flora, microorganisms, placenta, uterus, pregnancy, preterm labor, postpartum hemorrhage

## Abstract

Recent metagenomics and microbiology studies have identified microorganisms that are typical of the fetoplacental unit. Considering this emerging evidence, the placenta, uterus, and the amniotic cavity are not sterile and not immune privileged. However, there is evidence for a beneficial interaction between active maternal immune system and the presence of commensal pathogens, which lead to an immune-tolerant state, thereby preventing fetal rejection. Multiple conditions associated with the loss of the normal flora are described (dysbiosis), which could result in perinatal and puerperal adverse events, including, directly or indirectly, postpartum hemorrhage. Altered flora when associated with a severe proinflammatory state and combined with patient's genetic and environmental factors confers a high-risk adverse outcome. Better understanding of the adverse role of dysbiosis in pregnancy outcome will improve maternal outcome.

## Immunology of Pregnancy and the Role of Uterine and Placental Microbiota

In pregnancy, there is an increased predisposition toward infectious complications secondary to the transient and physiological immunocompromise. The amniotic cavity and choriodecidual junction have been traditionally described as immune-privileged entities, free of inflammatory response or microbiological growth. Nevertheless, the presence of infiltrating cells belonging to the maternal immune system in the choriodecidual region has been recently reported. Approximately 70% of decidual leukocytes are natural killer (NK) cells, 20 to 25% are macrophages, 3 to 10% are T cells, and 1.7% are dendritic cells. The described immune cell composition suggests that the rejection response that would be triggered by the implanted semi/allogeneic blastocyst, just like an allotransplant, is prevented. Paternal contribution has been considered as an exogenous component, translated to persistent immune tolerance to maternal cell infiltrates, thereby avoiding rejection processes (1). However, the theory of a decrease in immune response still remains controversial, especially because immune tolerance is necessary for a favorable pregnancy outcome. It is known that inactivation of NK cells could interfere with regulation of trophoblastic invasion contributing to abnormal placentation, which is a predisposing factor to postpartum hemorrhage (PPH) (2). Additionally, transduction of inhibitory factors involved in the inflammatory cascade such as FAS and FAS-L–associated ligands could result in miscarriage or stillbirth due to difficulties to maintain pregnancy (1). There are two proinflammatory processes related to pregnancy, predominantly represented by a T_H_1 response (it was previously considered to be absent during gestation and associated with adverse perinatal outcome). However, both the implantation process and labor involve, to some degree, inflammatory processes (1).

On the other hand, partial suppression of the T_H_2 response is required for fetal growth and development. Maternal dendritic cells that are found in the decidua present fetal antigens to maternal T lymphocytes seeking immunological tolerance. In contrast, in organ transplants, dendritic cells belonging to the transplanted organ present foreign antigens to the host's T lymphocytes (1). During this stage of gestation, anti-inflammatory cytokines are also synthesized to avoid adverse outcome. Regardless of the presence of decidual inflammatory cell infiltrates, trophoblastic development stimulates cytokine/chemokine release, such as [CXCL12, interleukin 18 (IL-18), and transforming growth factor β], promoting infiltration of immune system cells. This has been thought to improve pregnancy homeostasis, and any disturbance with this process could alter the pregnancy's outcome (3).

Additionally, recent advances in metagenomics have shown that the placenta harbors its own rich and diverse microbiota, which has been studied and described in healthy pregnancies (4). Altered placental microbiota (dysbiosis) induces a proinflammatory state leading to adverse maternal outcomes such as preterm labor, chorioamnionitis, premature rupture of membranes, intrauterine growth restriction, and even PPH (5).

The precise role of the uterine and placental microbiota in inducing immune tolerance to maternal and paternal antigens during gestation is still not clear. It is viewed that the microbiota works synergistically with the maternal inflammatory infiltrate and the trophoblast, preventing rejection (1). This likely related to the trophoblastic, macrophage, and decidual cell synthesis of the Toll-like receptor 4. This receptor induces interferon type 1 when exposed to microbial lipopolysaccharides that is present in the normal microbiota (1). Interferons are polypeptides that have 3 basic roles: (1) develop antimicrobial environment, (2) modulate the innate immune system, and (3) activate the adaptive immune system. In this way, the microbiota present in the maternal–fetal interface could be responsible for the basal expression of peptides, allowing modulation of the maternal immune system during fetal development and preventing the colonization by pathogenic microorganisms (1).

Yet, it is fundamental to recognize the existence of microorganisms that are present in fetal meconium and umbilical cord blood (6, 7). This confirms that the fetus is not sterile and that possibly the maternal microbiota is essential for development of the fetal microbiota, as well as acts as initiating stimulus for the fetal and neonatal innate immune system (4).

It becomes evident that the presence of microorganisms in these areas might not lead to adverse perinatal outcomes. In contrast, it could lead to the development of protective mechanisms to avoid such adverse outcomes. Different environmental or innate factors could alter the usual composition and function of microbiota during pregnancy. These processes allow pregnancy preservation and the normal uterine function during the immediate puerperium.

## Composition and Variations of Uterine, Placental, and Vaginal Microbiota

Generally, human microbiota's composition depends on its location, the host's genetic composition, dietary intake, and immune status (4). The female's reproductive system's microbiota changes according to her hormonal status during the menstrual cycle, as well as during the perigestational period (preconception and postconception) (4). Non-pregnant woman's vaginal microbiome has a great variety of microorganisms, with significant changes related to hormonal influx, age, and estrogen concentrations, which condition the acidity of the vaginal environment. This composition prevents the colonization of pathogens that do not belong to the vaginal milieu (5). More than 20 species of *Lactobacillus* species have been described, a dominant species in quantity as compared to the other commensal microorganisms. They oversee the production of lactic acid and compete for the vaginal wall surface (nutrients and cellular receptors), avoiding colonization by other pathogens. In a lesser proportion, there are also anaerobic organisms such as *Prevotella, Megasphaera, Gardnerella vaginalis, Sneathia*, and *Atopobium vaginae* (5).

The interaction between host microorganisms and those taking place among microorganisms start before birth and tend to vary among individuals especially due to their hormonal status. This leads to a high concentration of *Lactobacillus* species, *Propionibacterium* species, and Enterobacteriaceae presence during pregnancy in the healthy placental tissue (infection-free) (8–10). It is still unclear how microorganisms manage to enter the fetoplacental compartment. It is believed that the bacteria can ascend through the vagina and can be transported by antigen-presenting cells and carried into the amniotic cavity or colonize by hematogenous dissemination. The ascending path gains relevance considering the great portion of the vaginal microbiota that are incorporated in the placental microbiome are *Lactobacillus* species (5).

In a pregnant woman, it has been demonstrated that the composition of vaginal microbiota tends to fluctuate less than in a non-pregnant woman. During this period, *Lactobacillus* bacteria continue to prevail, but with the increase in four additional strains (*Lactobacillus crispatus, Lactobacillus jensenii, Lactobacillus gasseri*, and *Lactobacillus vaginalis*), leading to a decrease in anaerobic species. The increased estrogen levels in pregnant women lead to increased deposits of glycogen in the vaginal epithelium, which provides a better substrate for the growth of *Lactobacillus*, as well as the possibility for higher protective lactic acid production (5).

Several studies confirmed that during pregnancy *Lactobacillus* strain-specific vaginal microbiome could change according to the gestational age (11, 12). In a longitudinal study of the vaginal microbiota of 22 patients with non-complicated births, slight and occasional changes among diverse communities of *Lactobacillus* were found. However, the changes related to anaerobic bacteria predominance were rather rare (13). These changes were associated with lesser hormonal variability during gestation, decrease in sexual activity, or changes in production of vaginal secretions (5).

Microorganisms from the oral cavity and the gastrointestinal tract such as Enterobacteriaceae have been found in the placental environment. This has been associated with increased immune tolerance during pregnancy, which enables bacterial translocation from the gastrointestinal system to the bloodstream. This would create an access through the blood stream from different organ systems toward the uterine cavity (4).

## Placental Microbiota Behavior in Pregnancy With Adverse Outcomes

The microbial invasion (with non-commensal microorganisms) of the fetoplacental junction has been associated with maternal and neonatal morbidity. Studies showed the existence of different microorganisms in the placenta and the amniotic cavity with miscarriage, chorioamnionitis, premature rupture of the membranes, preterm birth, and stillbirth (14, 15). However, new evidence has also determined that the same type of pathogens might be present in births without associated complications. Therefore, genetic and/or environmental mechanisms may enable the progress of adverse perinatal outcomes due to the existence of the germs in a specific gestational stage (4). Alterations in the placental and amniotic microbiome can be associated with different pathologies during gestation, as well as can be associated with bacterial vaginosis (4).

Studies based on the analysis of nucleic acids of the placental tissue in pathologic pregnancies showed a predominance of anaerobic germs over beneficial *Lactobacillus*. The placental tissue analyzed in cases of preterm birth have found a higher number of *Prevotella, Bacteroides, Peptostreptococcus, Gardnerella, Mobiluncus*, and *Mycoplasma* species (16–19). *Streptococcus agalactiae, Fusobacterium nucleatum*, and *Ureaplasma parvum* species have been found in women with chorioamnionitis at a higher proportion (4). These microorganisms tend to have lower virulence outside the intrauterine environment. For example, *F. nucleatum* is an anaerobic microorganism that is generally located in the oral cavity mucosa. Nevertheless, the hematogenous spread of the same bacteria toward the placenta modifies the endothelial permeability of placental vasculature, which allows the entrance of other pathogenic organisms locally including *Escherichia coli* (5).

The analysis of oral bacteria such as *Fusobacterium* or *Capnocytophaga* in the placenta samples of women with preterm birth has been linked to periodontal disease that developed toward the end of gestation (20, 21). However, it is important to recognize that not only microorganisms from the oral cavity have been associated with these types of modified flora. As pregnancy progresses, there is a change in the gastrointestinal microbiota related to hormonal variations. Studies have shown dramatic changes in the composition of fecal flora of pregnant women from the first trimester until the third trimester, with increases in Proteobacteria and Actinobacteria content and decreases in *Lactobacillus* (5). This modification in colonizing germs may benefit from physiological changes in the maternal immune milieu, mostly becoming evident toward the end of the pregnancy. Such altered flora is translocated toward the bloodstream, reaching the amniotic and placental cavity, and creating a proinflammatory environment.

The ascent of vaginal colonizing pathogens to the uterine cavity has been etiologically linked with preterm birth. Hormonal and changing maternal environments lead to decrease in *Lactobacillus* species, allowing the colonization and access of pathogenic germs to the uterine cavity (5). A prospective cohort of 88 patients examining this association concluded that there is a correlation between microbial vaginal diversity and the progress to preterm birth (5). The pathogens that do not belong to the vaginal microbiota promote inflammatory mechanisms affecting the fetus and choriodecidual tissues. For example, secondary intra-amniotic infection due to unusual microorganisms triggers an inflammatory cascade associated with a large increase in the release of prostaglandins, metalloproteinases, and proinflammatory cytokines, which promote uterine activity leading to a major decrease in cervical collagen content (5).

## The Relation Between Placental and Uterine Microbiota and Postpartum Hemorrhage

The changes in the uterine and placental microbiota could determine an increase in PPH risk. After delivery, a state of acute postpartum myometritis with local inflammatory phenomena is seen, and the secondary dysbiosis causes muscular fatigue leading to PPH. This causality has been demonstrated in patients having significant hemorrhagic episodes of unclear etiology (22).

The most frequent cause of PPH is uterine atony (75% of cases). Multiple risk factors antepartum and intrapartum have been described for this clinical presentation (23), in those where it could be correlated with modification of the microbiota. For some of these risk factors, it is plausible and of clear etiology that explains the myometrial fiber dysfunction inability to contract. However, in many cases (up to 30% of the patients do not have risk factors), the phenomena could be secondary to local dysbiosis (22). Through immunofluorescence studies of uterine cavity samples, an increased association of inflammatory cells such as neutrophils, macrophages, and mastocytes was evidenced in patients with PPH of unclear etiology as compared to patients without associated hemorrhagic episodes. These immune cells are a principal source for chemical mediators exerting potent effect on the smooth vascular and uterine muscle. The accumulation of local uterine exudate that is associated with inflammatory response in interstitial spaces progresses to stromal edema and damage to uterine contractile function (22).

The term “acute postpartum myometritis” has been proposed to define local inflammation when there is no association with infection. Therefore, many theories related to the exposure of the maternal blood to amniotic fluid or fetal tissue have been suggested. This might promote the activation of the complement system, leading to increased neutrophil and macrophage activation and infiltration, as well as mastocyte degranulation in uterine and placental tissues, with the same deleterious effect on uterine contraction (22). Nevertheless, changes in the uterine microbiota and infiltration by pathogenic organisms (infection) may lead to the same response as described above. With this emerging insight, the local dysbiosis followed by activations in inflammatory cells and changes described in myometrial tissue could directly be associated with the development of PPH.

Similarly, Table 1 lists the diverse risk factors medium and high resulting in PPH as they possibly are related to the altered microbiota. Most of such direct causality has not been established yet. At this point, they remain presumptive based on the paucity of available evidence while it opens the possibility for pursuing new lines of clinical investigation.

**Table 1:** PPH risk factors and uterine microbiome alterations.

**Medium risk**	**Possibly related to dysbiosis**	**High risk**	**Possibly related to dysbiosis**
**ANTEPARTUM RISK FACTORS**			
Previous cesarean section, uterine surgery, or multiple laparotomy	Yes	Placenta previa (24, 25)	Yes
Multiple gestation	No data	Placenta accreta	Yes
>4 Previous births	No	Platelets <70,000	Yes
Uterine fibroids	Yes	Known coagulopathy	Yes
Fetus estimated <4,000 g	No	Abruptio placentae	Yes
Obesity high (body mass index >40 kg/m^2^)	No	>2 Intermediate risk factors	Factor dependent
Hematocrit <30% plus another risk factor	No data	–	–
**INTRAPARTUM RISK FACTORS**			
Chorioamnionitis	Yes	Active vaginal bleeding	Yes
Oxytocin drip during >24 h	Yes	Preeclampsia with severity criteria	Yes
Prolonged second stage of labor	Yes	≥2 Intermediate risk factors antepartum/intrapartum	Factor dependent
Use of magnesium sulfate	No	–	–

For some risk factors, the relationship with the altered microbiota is better defined. Maternal obesity or overweight is directly related to inflammatory mechanisms active in placental tissue, conditioning dysbiosis, and anomalies regarding fetal well-being (4). The increase in adipose tissue and the associated peripheral insulin resistance lead to an increase in cytokine levels that act directly to increase pathology severity (5). For instance, in diabetic patients, a decrease in *Acinetobacter* load in the gastrointestinal tract (a commensal organism in this location) was coupled with a decrease in eosinophilic levels and reduced placental anti-inflammatory gene expression, including IL-10 (4). These potential pathological mechanisms are currently being further investigated (26).

Acute and chronic abruptio placentae cause significant maternal and perinatal morbidity. However, at present, it is frequently difficult to establish a direct correlation because of the absence of appropriate placental monitoring (27). However, the clinical manifestations with altered patterns of uterine contractility and the increase in incidence of preterm birth cases, premature rupture of membrane, and preeclampsia support the hypothesis of a contribution by the presence of dysbiosis as an underlying physiopathology.

The most accepted hypothesis for placenta accreta etiology states that it is a spectrum of endometrial interface imperfection that leads to failed decidualization in the area of uterine scar, allowing an abnormal and profound anchoring of the villous trophoblast. What is the role of the microbiota in these abnormal implantation phenomena? That is a question that has not been addressed yet. However, there is evidence for the presence of proinflammatory biomarkers that identify such increased risk, which suggest involvement of dysbiosis in the placenta and amniotic cavity in patients with placenta accreta (28).

## Conclusion

Microbiological load, typical of the human being, turns out to be beneficial and necessary in all areas in the body. Nevertheless, until recently, the amniotic cavity and the fetoplacental unit were considered as an immune-privileged area and free of associated inflammatory processes. The current evidence indicates that inflammatory mechanisms and the immunotolerance together are both necessary and coexist. It is possible that modification of the beneficial microbiota that is required for maintaining pregnancy leads to immune intolerance. Such enables development of a proinflammatory state leading to preterm birth, premature rupture of membranes, stillbirth, and PPH. It is very important to broaden the microbiology and metagenomics investigations that allow recognizing the high impact of these types of associated phenomena, especially PPH, recognized worldwide to be associated with high lethality. Possibly by avoiding development of maternal dysbiosis, this huge clinical burden could be reduced.

## Author Contributions

ME, JB, and MH: contribution to the conception of the work, intellectual production, writing of the manuscript and critical revision, final approval of the manuscript, agreement to be accountable for all aspects of the work, research of information.

### Conflict of Interest

The authors declare that the research was conducted in the absence of any commercial or financial relationships that could be construed as a potential conflict of interest.
